# Eicosapentaenoic Acid Level Predicts Long-Term Survival and Cardiovascular or Limb Event in Peripheral Arterial Disease

**DOI:** 10.3400/avd.oa.23-00079

**Published:** 2024-03-08

**Authors:** Hisao Kumakura, Ryuichi Funada, Yae Matsuo, Toshiya Iwasaki, Kuniki Nakashima, Eitoshi Tsuboi, Shuichi Ichikawa

**Affiliations:** 1 Department of Cardiovascular Medicine, Cardiovascular Hospital of Central Japan (Kitakanto Cardiovascular Hospital), Shibukawa, Gunma, Japan; 2 Department of Cardiovascular Surgery, Cardiovascular Hospital of Central Japan (Kitakanto Cardiovascular Hospital), Shibukawa, Gunma, Japan

**Keywords:** eicosapentaenoic acid, limb events, all-cause mortality, major adverse cardiovascular event, peripheral arterial disease

## Abstract

**Objectives:** We examined the relationship between plasma eicosapentaenoic acid (EPA) level and long-term all-cause death (ACD) and cardiovascular or limb events in patients with peripheral arterial disease (PAD).

**Method:** We performed a prospective cohort study on 637 PAD patients. The endpoints were ACD, major adverse cardiovascular events (MACEs), and lower extremity arterial events (LEAEs).

**Results:** The incidences of ACD, MACEs, and LEAEs had correlation with EPA levels (p <0.05). Plasma EPA level had significant positive correlations with high-density lipoprotein cholesterol, triglyceride, and estimated glomerular filtration rate (eGFR), and negative correlation with C-reactive protein (CRP). In Cox stepwise multivariate analysis, lower EPA (hazard ratio [HR]: 0.996, 95% confidence interval [CI]: 0.993–1.000, p = 0.034), ankle brachial pressure index (ABI), body mass index, serum albumin, eGFR, age, CRP, D-dimer, critical limb ischemia, diabetes, cerebrovascular disease (CVD), and statin were related to ACD (p <0.05); lower EPA (HR: 0.997, 95% CI: 0.994–1.000, p = 0.038), ABI, serum albumin, eGFR, age, diabetes, coronary heart disease, CVD, and statin were related to MACEs (p <0.05); and lower EPA (HR: 0.988, 95% CI: 0.982–0.993, p <0.001), ABI, and low-density lipoprotein cholesterol were related to LEAEs (p <0.05).

**Conclusions:** Low plasma EPA level was a significant risk factor for ACD, MACEs, and LEAEs in patients with PAD.

## Introduction

The ω-3 polyunsaturated fatty acids (PUFAs), including eicosapentaenoic acid (EPA) and docosahexaenoic acid (DHA), have significant anti-inflammatory and anti-atherosclerotic actions.^1^^,^^2)^ The Japan EPA lipid intervention study (JELIS) trial has reported the preventive effects of EPA on coronary heart disease (CHD), and EPA aids in reducing the levels of triglyceride in patients with hypercholesterolemia.^3)^ Furthermore, PUFAs play an important role in resolving inflammation as results of reducing inflammation markers such as interleukin-6, tumor necrosis factor-alpha (TNF-α), and interleukin-1β.^4)^ TNF-α relates to peripheral arterial restenosis, cardiovascular dysfunction, and cardiovascular events.^5^^,^^6)^

The subanalysis of the JELIS trial has showed that EPA reduced the incidence of recurrent cerebrovascular disease (CVD).^7)^ Moreover, the incidence of CHD is also reduced in patients with peripheral arterial disease (PAD).^8)^ Patients with PAD have severe atherosclerosis that causes poor prognosis due to CHD and CVD.^9^^,^^10)^ PAD patients were more likely to have low basal EPA and DHA levels or EPA/arachidonic acid (AA) ratio.^11^^,^^12)^ Several studies have reported that low EPA level and EPA/AA ratio are atherosclerotic biomarkers reflecting the risk of limb events in PAD patients in small numbers and/or retrospective studies.^13^^–^^15^)

However, long-term pure limb events and cardiovascular or cerebrovascular events associated with basal plasma EPA levels have not been investigated in prospective study in patients with PAD. Moreover, long-term all-cause mortality based on plasma EPA levels has not been examined in patients with PAD. Therefore, the aim of the current study was to assess these outcomes for 15 years associated with basal plasma EPA levels in patients with PAD.

## Patients and Methods

### Patients

The subjects were Japanese patients with PAD who were referred to the Cardiovascular Hospital of Central Japan in the period from March 1, 2002 to January 31, 2023. The methods and design of the prospective study were conducted in accordance with the Declaration of Helsinki, and the protocol used in this research was approved by the Medical Ethics Committee in our institution (CCJ-EA-006). Patients who gave written informed consent to participation in the study and met the following inclusion criteria were selected: (1) ankle brachial pressure index (ABI) <0.90; (2) clinical symptoms (critical limb ischemia [CLI] or intermittent claudication); and (3) a stenotic lesion of ≥70% in the femoropopliteal or iliac artery on ultrasound or angiography. Patients with peripheral lesions owing to non-atherosclerotic causes were excluded. Patients treated with EPA curatives or ingested dietary EPA-rich supplements at baseline and during follow-up period were excluded. Patients with dementia were also excluded on account of the difficulty in checking medical status and vital signs.

### Baseline clinical characteristics

Age, ABI, body mass index (BMI), and smoking history status were obtained for each patient. A morning blood sample was collected to determine the levels of albumin, glucose, creatinine, triglyceride, total cholesterol, high-density lipoprotein cholesterol (HDL-C), low-density lipoprotein cholesterol (LDL-C), C-reactive protein (CRP), and D-dimer. Moreover, we collected a methylated blood sample for PUFAs measure. The measurements of serum EPA, DHA, and AA were performed using a Shimadzu GC-2010 gas chromatograph (Shimadzu Corporation, Kyoto, Japan). Diabetes mellitus (DM) was specified as a fasting plasma glucose level >126 mg/dL in at least two measurements or receiving antidiabetes therapy. Hypertension was defined as required antihypertensive oral therapy or blood pressure ≥140/90 mmHg recorded at least twice.

### Data analysis and endpoints

Each subject was followed up at 2-, 4-, or 6-month intervals after treatment. Medical status and vital signs were assessed using hospital data and written questionnaires on life status monitored by the Foot Care Club.^10^^,^^16)^ Ischemic cerebral infarction was determined as the presence of a new focal neurological deficit, with brain computed tomography or magnetic resonance imaging to determine the focal lesions. Transient ischemic attack (TIA) was defined as the presence of a new neurological symptom lasting <24 h. The diagnosis of myocardial infarction was that used previously.^16^^,^^17)^ Limb events during follow-up were defined as the progression of a new stenosis or restenosis identified as a decrease in ABI of ≥0.15 and ≥50% stenosis on duplex ultrasonography or angiography in the lower extremity arterial lesions and above-the-ankle amputation.^16^^,^^18)^

The endpoints were all-cause death (ACD), major adverse cardiovascular events (MACEs: ACD, TIA, non-fatal CVD, or non-fatal myocardial infarction), and lower extremity arterial events (LEAEs: repeat revascularization for a lower extremity artery, occurrence of a new lower extremity lesion, or major amputation).

### Statistical analysis

IBM SPSS Statistics ver. 25.0 (IBM Corp, Armonk, NY, USA) was utilized for all statistical analyses. Categorical variables are expressed as a number (%) and were compared via chi-square test. Continuous variables are shown as a median (interquartile range) and were evaluated with the Mann–Whitney U test. In multiple regression analysis, all risk factors were first examined with simple Pearson correlations. Factors with p <0.05 in this correlation analysis were used in stepwise forward multiple regression analysis to define relationships between EPA level and individual risk factors. Kaplan–Meier analysis was used to evaluate ACD, MACEs, and LEAEs with comparison by the log-rank test. In the Cox multivariate regression model, hazard ratios (HRs) and 95% confidence intervals (CIs) were estimated by means of forward stepwise regression analyses with Wald statistic to define significant factors associated with endpoints. The Cox univariate regression model was used for individual factors to search for affecting factors for endpoints. A p <0.05 was determined as statistically significant.

## Results

### Patient characteristics and causes of death

Among 658 subjects, follow-up was possible for 637 patients, and the median and mean ages were 73 (67–80) and 72.8 ± 10.3 years. The median follow-up periods were 71 (34–126) months. There were 235 deaths (36.9%) during the follow-up period. The cumulative 5-, 10-, and 15-year freedom from ACD in all patients were 79.6%, 60.4%, and 47.7%, respectively. The prevalence of cardiovascular-related death was 53.2% (n = 125), as cardiac or major vascular disease (n = 103, 43.8%) and CVD (n = 22, 9.4%). Other causes of deaths were malignancy (n = 53, 22.6%), pneumonia (n = 34, 14.5%), and other causes (n = 23, 9.8%).

The median and mean EPA levels were 50.9 (32.4–80.0) and 62.6 ± 44.8 μg/mL. Following these results, the patients were divided into two groups based on plasma EPA levels with median: E1: ≤50.9 μg/mL (n = 319) and E2: ≥51.0 μg/mL (n = 318). The comorbidities and clinical characteristics in PAD patients divided by median are shown in **Table 1**.

**Table:** Table 1 Characteristics of subjects in all patients, E1, and E2 based on EPA levels

	All Patients (n = 637)	E1 EPA ≤ 50.9 μg/mL (n = 319) (50.1%)	E2 EPA ≥ 51.0 μg/mL (n = 318) (49.9%)	p-value
Age (year)	73 (67–80)	73 (66–81)	74 (67–80)	0.575
Male sex	464 (72.8%)	231 (72.4%)	233 (73.3%)	0.859
ABI	0.71 (0.55–0.86)	0.70 (0.50–0.84)	0.72 (0.58–0.87)	0.015
Intermittent claudication	543 (85.2%)	263 (82.4%)	280 (88.1%)	0.057
Critical limb ischemia	94 (14.8%)	56 (17.6%)	38 (11.9%)	0.057
Body mass index (kg/m^2^)	22.4 (20.3–24.5)	21.8 (19.6–24.4)	22.7 (20.9–24.6)	0.002
Coronary heart disease	229 (35.9%)	121 (37.9%)	108 (34.0%)	0.322
History of stroke or TIA	97 (15.2%)	52 (16.3%)	45 (14.2%)	0.508
Diabetes mellitus	287 (45.1%)	147 (46.1%)	140 (44.0%)	0.633
Hypertension	457 (71.7%)	230 (72.1%)	227 (71.4%)	0.861
Smoking	478 (75.0%)	241 (75.5%)	237 (74.5%)	0.784
Medications				
Aspirin	420 (65.9%)	205 (64.3%)	215 (67.6%)	0.372
Thienopyridines	268 (42.1%)	132 (41.3%)	136 (42.8%)	0.723
Cilostazol	93 (14.6%)	46 (14.4%)	47 (14.8%)	0.911
Beraprost	202 (31.7%)	108 (33.9%)	94 (29.6%)	0.709
ARB	263 (41.3%)	131 (41.4%)	132 (41.5%)	0.936
Ca antagonist	342 (53.7%)	170 (53.3%)	172 (54.1%)	0.874
β-blocker	103 (16.2%)	62 (19.4%)	41 (12.9%)	0.031
Statin	467 (73.3%)	238 (74.6%)	229 (72.0%)	0.459
Revascularization	342 (53.8%)	152 (47.8%)	190 (59.7%)	0.003
Basic metabolic panel				
EPA (μg/mL)	50.9 (32.4–80.0)	32.4 (22.9–40.6)	80.7 (63.3–110.5)	<0.001
AA (µg/mL)	144.7 (115.7–177.9)	137.5 (109.6–170.4)	150.5 (125.6–186.0)	<0.001
DHA (µg/mL)	114.7 (85.0–158.9)	92.4 (72.5–115.0)	151.5 (114.5–195.1)	<0.001
EPA/AA ratio	0.35 (0.22–0.55)	0.23 (0.16–0.31)	0.55 (0.40–0.80)	<0.001
Serum albumin (g/dL)	4.0 (3.7–4.3)	3.9 (3.7–4.2)	4.1 (3.9–4.3)	<0.001
CRP (mg/dL)	0.15 (0.07–0.39)	0.17 (0.08–0.47)	0.14 (0.07–0.33)	0.134
D-dimer (µg/dL)	0.8 (0.5–1.7)	1.0 (0.5–2.3)	0.7 (0.5–1.2)	<0.001
eGFR (mL/min/1.73 m^2^)	59.4 (44.1–72.0)	57.6 (38.1–71.0)	60.5 (49.1–73.1)	<0.001
Total cholesterol (mg/dL)	191 (163–218)	184 (157–210)	197 (172–226)	<0.001
Triglyceride (mg/dL)	136 (99–191)	126 (95–177)	147 (102–195)	0.005
HDL-C (mg/dL)	50 (42–52)	49 (41–58)	52 (44–65)	0.005
LDL-C (mg/dL)	113 (90–133)	110 (86–94)	117 (94–140)	0.002

EPA: eicosapentaenoic acid; ABI: ankle brachial pressure index; TIA: transient ischemic attack; ARB: angiotensin receptor blocker; AA: arachidonic acid; DHA: docosahexaenoic acid; CRP: C-reactive protein; eGFR: estimated glomerular filtration rate; HDL-C: high density lipoprotein cholesterol; LDL-C: low density lipoprotein cholesterol

Patients in the E1 category had lower ABI, BMI, serum albumin, estimated glomerular filtration rate (eGFR), levels of total cholesterol, triglyceride, LDL-C, HDL-C, AA, DHA, EPA/AA ratio, and higher D-dimer. The prevalence of revascularization was lower, and treatment with β-blocker was higher in the E1 category.

### Correlational statistics among EPA and cardiovascular risk factors

Plasma EPA level had significant positive correlations with ABI, serum albumin, eGFR, total cholesterol, triglyceride, and HDL-C, and negative correlations with CLI, CRP, D-dimer, and CHD in simple Pearson correlation analysis (p <0.05). Within PUFAs, plasma EPA level had significant positive correlations with DHA level and EPA/AA ratio in simple Pearson correlation analysis (p <0.01). The plasma EPA levels had significant positive correlations with HDL-C, triglyceride, and eGFR, and negative correlation with CRP in correlational statistics among these significant factors with stepwise forward multiple regression analysis (p <0.05, **Table 2**).

**Table:** Table 2 Correlational statistics between eicosapentaenoic acid level and other risk factors

Factor	β	B	95% CI	p-value
HDL-C (mg/dL)	0.152	0.431	0.197 to 0.665	<0.001
Triglyceride (mg/dL)	0.131	0.057	0.021 to 0.092	0.002
C-reactive protein (mg/dL)	–0.098	–3.395	–6.111 to –0.679	0.014
eGFR (mL/min/1.73 m^2^)	0.102	0.180	0.040 to 0.319	0.012

R^2^ = 0.058, F for change in R^2^ = 9.334, p <0.001. β: standardized coefficient; B: non-standardized coefficient; CI: confidence interval for B; HDL-C: high-density lipoprotein cholesterol; eGFR: estimated glomerular filtration rate

### Factors for ACD, MACEs, and LEAEs

**Figure 1:**
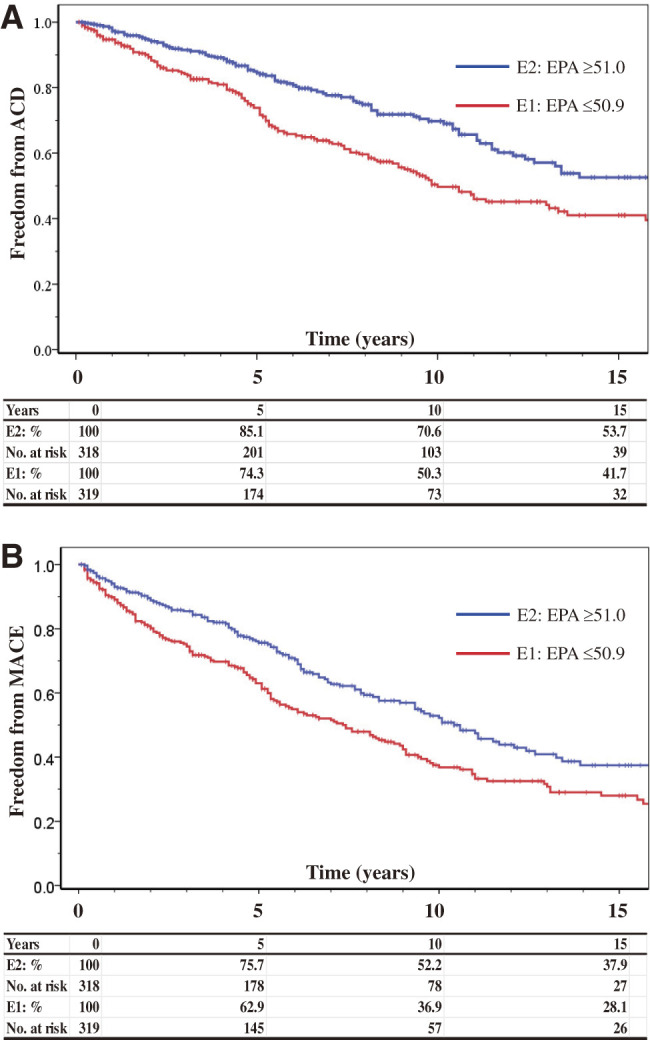
Fig. 1 (**A**) Freedom from ACD according to EPA levels is demonstrated with significant difference between E1 and E2 (p = 0.003). (**B**) Freedom from MACEs according to EPA levels is demonstrated with significant difference between E1 and E2 (p = 0.001). ACD: all-cause death; EPA: eicosapentaenoic acid; MACEs: major adverse cardiovascular events.

The 15-year cumulative rates for freedom from ACD are indicated in **Fig. 1A**. There was significant difference between E1 and E2 (p = 0.003). In Cox forward stepwise regression analysis, age, CRP, D-dimer, lower EPA, ABI, BMI, serum albumin, eGFR, CLI, DM, and CVD were related to ACD, and statin treatment decreased ACD (p <0.05, **Table 3**).

**Table:** Table 3 Cox stepwise multivariate regression analyses for ACD, MACEs, and LEAEs

Factors	ACD			MACEs			LEAEs		
	Multivariate analysis			Multivariate analysis			Multivariate analysis		
	HR	95% CI	p-value	HR	95% CI	p-value	HR	95% CI	p-value
Age (year)	1.071	1.055–1.087	<0.001	1.038	1.025–1.051	<0.001			
ABI	0.558	0.327–0.952	0.032	0.565	0.368–0.867	0.009	0.373	0.212–0.657	0.001
Body mass index (kg/m^2^)	0.939	0.897–0.983	0.007						
Critical limb ischemia	1.596	1.097–2.322	0.014						
Coronary heart disease				2.249	1.778–2.845	<0.001			
History of stroke or TIA	1.478	1.052–2.076	0.024	1.460	1.089–1.959	0.011			
Diabetes mellitus	1.426	1.054–1.929	0.021	1.403	1.114–1.767	0.004			
EPA (μg/mL)	0.996	0.993–1.000	0.034	0.997	0.994–1.000	0.038	0.988	0.982–0.993	<0.001
Serum albumin (g/dL)	0.435	0.321–0.590	<0.001	0.509	0.387–0.670	<0.001			
CRP (mg/dL)	1.099	1.014–1.192	0.022						
D-dimer (µg/dL)	1.054	1.030–1.079	<0.001						
eGFR (mL/min/1.73 m^2^)	0.987	0.982–0.993	<0.001	0.994	0.989–0.999	0.017			
LDL-C (mg/dL)							1.005	1.001–1.010	0.028
Statin	0.486	0.367–0.645	<0.001	0.534	0.423–0.675	<0.001			

HR: hazard ratio; CI: confidence interval; ABI: ankle brachial pressure index; TIA: transient ischemic attack; EPA: eicosapentaenoic acid; CRP: C-reactive protein; eGFR: estimated glomerular filtration rate; LDL-C: low-density lipoprotein cholesterol; ACD: all-cause death; MACEs: major adverse cardiovascular events; LEAEs: lower extremity arterial events

The 15-year cumulative rates for freedom from MACEs are indicated in **Fig. 1B**. There was significant difference between E1 and E2 (p = 0.001). In Cox forward stepwise regression analysis, age, lower EPA, ABI, serum albumin, eGFR, CHD, CVD, and DM were related to MACEs, and statin decreased MACEs (p <0.05, **Table 3**).

**Figure 2:**
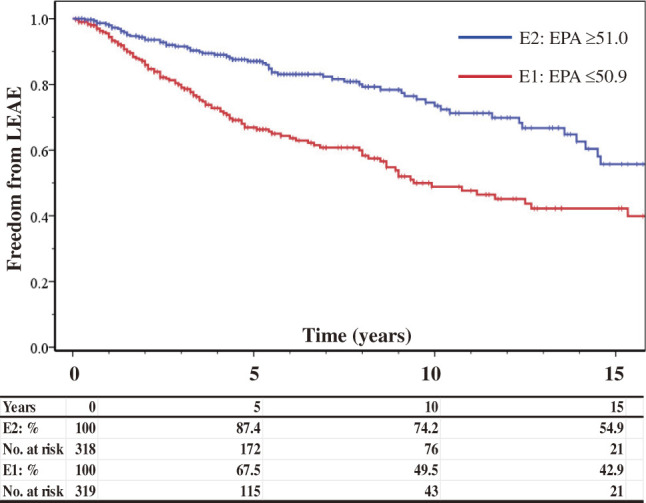
Fig. 2 Freedom from LEAEs according to EPA levels is demonstrated with significant difference between E1 and E2 (p <0.001). LEAEs: lower extremity arterial events; EPA: eicosapentaenoic acid.

The 15-year cumulative rates for freedom from LEAEs are indicated in **Fig. 2**. There was significant difference between E1 and E2 (p <0.001). In Cox forward stepwise regression analysis, lower EPA, ABI, and higher LDL-C were related to LEAEs (p <0.05, **Table 3**).

## Discussion

This study represented the first prospective clinical results for the relationships between basal plasma EPA level and 15-year ACD, MACEs, and LEAEs in patients with PAD. Compared with low plasma EPA level, patients with high plasma EPA level had significantly low rate in LEAEs during the follow-up period in PAD patients. The cumulative incidences of ACD and MACEs were also significantly increased relative to the lower plasma EPA levels. PUFAs play an important role in resolving inflammation as the result of reducing inflammation markers.^4)^ The subanalysis of the JELIS trial has reported that administration of highly purified EPA reduced the incidences of CHD and recurrent stroke in patients with hypercholesterolemia.^7^^,^^8)^

In this study, plasma EPA levels had significant positive correlations with HDL-C, triglyceride, and eGFR. High-density lipoprotein is a heterogeneous population of particles, and smaller HDL-C particles are more efficient in cholesterol efflux than large particles in macrophages.^19)^ Dense and smaller HDL-C particles have more anti-inflammatory and antioxidant properties reducing LDL-C oxidation in the subendothelial layer of the arterial wall.^20)^ PUFAs are effective in the reduction of cardiovascular risk associated with size changes and improvement in HDL-C particles.^21)^ In the current study, LDL-C is an independent risk factor for LEAEs. High LDL-C is an independent risk factor for patients with atherosclerotic vascular diseases, and intensive lowering therapy for LDL-C is effective for decreasing cardiovascular events in these patients.^22)^

We have reported obesity paradox and the geriatric nutritional risk index are significant predictors for ACD, MACEs, and MACEs plus limb events (MACLE) in PAD patients.^10^^,^^23)^ Plasma triglyceride levels have significant positive correlation with BMI.^10)^ In this study, lower BMI and serum albumin were related to ACD. Thus, malnutrition based on low BMI or triglyceride and chronic kidney disease are strong risk factors for ACD in patients with cardiovascular disease.^10^^,^^24^^,^^25)^ Moreover, increasing levels of PUFAs in membranes affect the uptake and intracellular metabolism of PUFAs in the kidney.^26)^ These results suggested that higher triglyceride and eGFR levels might be the causes of higher plasma EPA levels.

Higher CRP is a significant predictor for ACD, MACEs, and MACLE in PAD patients.^9^^,^^27)^ In the current study, the plasma EPA levels had significant negative correlation with CRP and the higher CRP levels significantly related to ACD. Thus, these inflammations induced by various cytokines affect both atherosclerosis development and cardiovascular remodeling in these subjects.^4^^,^^5)^

DHA is one type of PUFAs and improve endothelial dysfunction to increase the expression of endothelial nitric oxide synthase.^2)^ In this study, Kaplan–Meier analysis showed a significant difference only in the LEAEs divided into two groups based on DHA levels with median. Cox univariate analysis showed that DHA was a predictor for LEAEs. In stepwise multivariate analysis, DHA was not a significant predictor for LEAEs. However, DHA was a significant predictor for LEAEs if EPA was excluded from the factors in stepwise multivariate analysis.

Several studies have reported that high plasma EPA/AA ratio is an atherosclerotic biomarker reflecting the risk of LEAEs and MACEs in patients with PAD.^13^^,^^14)^ The reduced risks associated with high EPA/AA ratio could be ascribed to either a greater role of EPA in stabilizing atherosclerotic plaque or a smaller role of AA in destabilizing atherosclerotic plaque.^28)^ In the current study, Kaplan–Meier analysis showed significant differences in the LEAEs and MACEs divided into two groups based on EPA/AA ratio with median. However, Cox univariate analysis showed that EPA/AA ratio was a predictor for LEAEs alone. In stepwise multivariate analysis, EPA/AA ratio was not a predictor for LEAEs, suggesting a more meaning role for basal plasma EPA levels.

The results of **Table 3** and Kaplan–Meier curves in ACD and MACEs were relatively similar, but the result of LEAEs showed a different pattern. In this study, we have changed the outcomes from classical MACLE to LEAEs. In LEAEs, reduplicated cardiovascular events and ACD were removed from the outcome in MACLE, and the effect of EPA and other risk factors for pure leg events was articulated. Moreover, the DHA level and EPA/AA ratio had significant positive correlations with EPA level within PUFAs. As described above, the DHA level and EPA/AA ratio had not significant but similar effects for LEAEs alone. The overlapped effect of these three factors may be another cause of the different patterns in LEAEs compared with ACD or MACEs.

These results suggested that the administration of highly purified EPA or EPA plus DHA might be effective in reducing the incidence of ACD, MACEs, and LEAEs in PAD patients with low basal plasma EPA levels.

### Study limitations

There are several limitations in the current study. First, the study was based on data from a single facility and the number of patients was relatively small. Second, the prevalence of therapy with statins during the follow-up period was relatively low based on the recent guidelines, but the usage has increased over time. Further prospective long-term clinical follow-up data are needed for investigation of outcomes and risk factors with a larger patient cohort in PAD patients.

## Conclusion

Cumulative incidences of ACD, MACEs, and LEAEs had significant correlations with lower plasma EPA levels. Thus, low plasma EPA level was defined as a predictor for 15-year mortality, cardiovascular events, and fate of the limb in patients with PAD.

## Disclosure Statement

The authors declare no conflict of interest.

## Author Contributions

Study conception: HK and SI

Data collection: RF, YM, TI, KN, and ET

Analysis: HK and RF

Investigation: HK, RF, and SI

Writing: HK and SI

Funding acquisition: None

Critical review and revision: all authors

Final approval of the article: all authors

Accountability for all aspects of the work: all authors.
